# F18-FDG PET/CT Scanning in Angiosarcoma: Report of Two Cases

**DOI:** 10.4274/MIRT.020397

**Published:** 2011-08-01

**Authors:** Emel Tokmak, Elgin Özkan, Sule Yağcı, K. Metin Kır

**Affiliations:** 1 Ankara University Medical Faculty, Department of Nuclear Medicine, Ankara, Turkey

**Keywords:** Sarcoma-angiosarcoma, Positron emission tomography, F18 fluorodeoxyglucose, treatment monitoring

## Abstract

Angiosarcomas are uncommon tumors and constitute less than 5% of all soft tissue sarcomas. They are aggressive tumors with poor prognosis, therefore, it is quite important to determine disease extension and detect local recurrence and/or distant metastases for appropriate therapy management. In this paper, we aimed to demonstrate the potential role of 1F18-FDG PET/CT imaging by reporting two cases with angiosarcoma.

**Conflict of interest:**None declared.

## INTRODUCTION

Angiosarcomas are uncommon malignant tumors of vascular and lymphatic endothelium and constitute less than 5% of all soft tissue sarcomas. They are generally localized in lower extremities or in solid organs. The etiopathogenesis is still unclear; however, there are some declared theories such as presence of chronic edematous extremity, underlying trauma and radiotherapy history (1,2,3,4,5). Angiosarcomas are aggressive tumors with poor prognosis, therefore, it is quite important to determine disease extension and detect local recurrence and/or distant metastases for appropriate therapy management. 

There are some studies that reported the use of FDG PET and PET/CT in angiosarcomas (hepatic, cardiac, venous and cutaneous angiosarcomas) in the literature (1,6,7,8,9,10,11). In this paper, we aimed to report the potential benefits of F18-FDG PET/CT imaging in two angiosarcoma patients.

## CASE REPORT

**Patient 1**

A 56 year-old male patient with several palpable nodules on the medial side of his right leg and thigh underwent excisional biopsy and histopathological examination which revealed angiosarcoma. He was referred to our clinic for primary staging with -F18-FDG PET/CT. PET/CT images demonstrated multiple increased F18-FDG uptake regions along the medial side of right leg within the subcutaneous tissue (Figure 1A) and mildly increased 18-FDG uptake in both external iliac and inguinal lymph nodes. He received hyperthermic chemoperfusion therapy. One month later, therapy response was assessed by PET/CT. PET scan demonstrated the absence of FDG uptake in external iliac and inguinal lymph nodes and decreased but persisting uptake on the medial side of right leg (Figure 1B). After 4 courses of chemotherapy, third PET/CT scan showed significantly decreased uptake considered as favourable therapy response by the clinician (Figure 1C). The patient was followed with no additional treatment and no recurrence was observed in the two-year follow up period. 

**Patient 2**

A 47 year-old male patient with a history of angiosarcoma, suffered from swelling and increased heat in the left leg. Clinical examination showed subcutaneous nodules in the left leg and he was given one course of hyperthermic chemoperfusion therapy considering recurrent disease. PET/CT scan was performed to rule out distant metastasis after a week of hyperthermic chemoperfusion therapy. Whole body and lower extremity views were obtained. PET/CT of lower extremity revealed pathological uptake in inguinal area extending to medial side of the knee (Figure 2A) and non-homogenous FDG uptake at the left lower side of anterior abdominal wall which was considered as secondary to the previous chemoperfusion therapy (Figure 2B). According to PET/CT findings, there was no distant metastasis and he underwent wide surgical resection of the involved areas and recurrent disease was confirmed by histopathological examination. 

## LITERATURE REVIEW AND DISCUSSION

Angiosarcomas are aggressive tumors and local recurrence or distant metastases may develop in 30-35% of the patients after therapy (12). Chest, abdominal wall and retroperitoneal regions are the most frequent localizations of distant metastases. They are more frequently seen at advanced age and in men compared to women (5,13). 

Angiosarcomas have high proliferation rate and total excision of the tumor can be applicable in less than half of the patients. High mitotic activity and tumor necrosis are the main indicators of poor prognosis (7); therefore, the most adequate treatment approach is still wide excisional resection of the tumor. Additionally, some studies reported the limitations of neoadjuvant treatment modalities and concluded that chemotherapy/radiotherapy should be applied when complete excision is not possible. Response to therapy is variable considering the differences in tumor size, degree of infiltration, histopathologic subtype and tumor differentiation. However, total excision still can be feasible and improve the survival when the tumor is diagnosed at an early stage (2,7).

To our knowledge there are only a few reports about angiosarcomas at uncommon localizations (cardiac, hepatic and venous regions) detected by FDG PET and PET/CT (1,7,8,9,10) and the value of FDG PET in diagnosis and staging was well-defined in these studies. However, early detection of local and/or distant metastases is a challenging problem in high-risk patients and has an important role to predict the patients’ outcomes. The sensitivity and specificity of FDG PET and MRI for detecting local recurrences was found 73.7% and 94.3%; 88.2% and 96% in a study, respectively (12). Although MRI imaging seems to be more sensitive in this patient group, it could not be applied to patients with metallic implants. Edema and/or inflammatory changes at post treatment period could be responsible for false negative or positive results. It is also reported that FDG PET was considerably successful in demonstrating extra-pulmonary metastases (12). In another study, PET/CT was found to be more valuable in detecting local and/or distant metastases than primary staging in soft tissue sarcoma patients (8). In our second patient with recurrent disease, PET/CT was carried-out to rule out distant metastasis. Concurrent CT and/or MRI were not performed in this case. According to PET/CT findings, there was no distant metastasis. However, increased local metabolic activity in primary lesion site which is provided local recurrence by histopathological examination was detected. 

In a study with 42 soft tissue sarcoma patients, the reduction in glycolitic activity was found more accurate than size-based changes in the prediction of histopathological response to therapy (15). In another study of patients with soft tissue sarcoma, changes in FDG uptake was found to correlate with histopathological response, risk of tumor recurrence and survival (16). In our first case, F18-FDG PET/CT was used for both primary staging and therapy response evaluation. The number of lesions and SUVmax values were significantly decreased which is probably related with histopathological response as well the clinical follow up results. 

Considering these two patients, FDG PET/CT can be a promising imaging tool in angiosarcomas. It enables to detect disease extension and multiple tumor foci at a single session and is cost-effective by providing morphologic and functional images concurrently. PET/CT findings also might be useful in the prediction of histopathological response or unresponsiveness. Although PET/CT is not the first-line imaging tool in soft tissue sarcomas, its ability to detect local and/or distant metastases should be taken into account. Further studies with larger number of patients also should be carried-out to establish the association of SUV values with tumor aggressivity.

## Figures and Tables

**Figure 1a:**
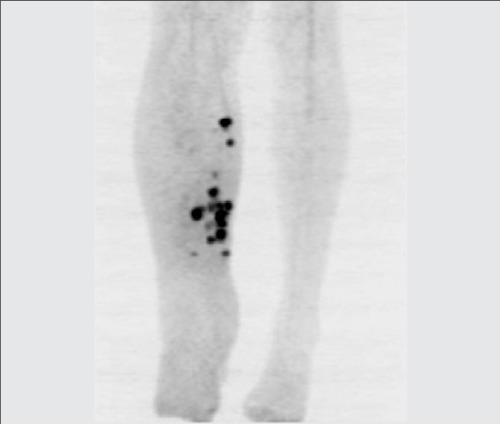
Maximum intensity projection (MIP) image of lowerextremity showed multiple increased activity (SUV_max_: 10.8) in thesubcutaneous tissue

**Figure 1b-c:**
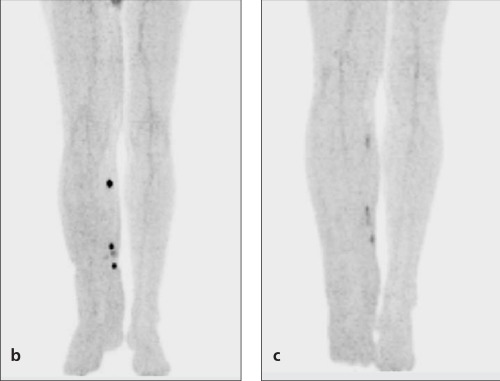
Follow up PET/CT images showed the significantdecrease in the amount of FDG uptake (1B;SUV_max_:5.2) and the firstyear examination after surgery and chemotherapy revealed favorableresponse to therapy ( 1C; SUV_max_: 2.8)

**Figure 2a:**
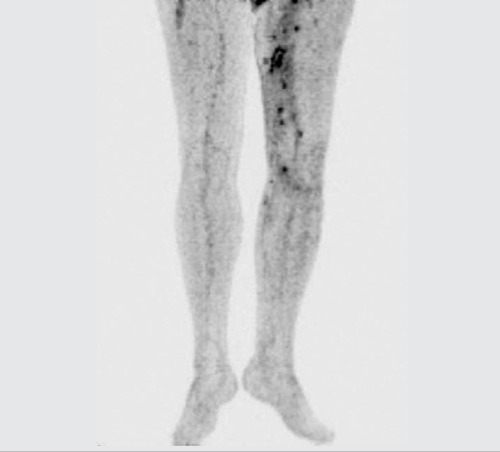
MIP image of lower extremity showed multiple foci ofFDG uptake (SUV_max_:6.6) on the left thigh and the medial side ofthe knee within the subcutaneous tissue

**Figure 2b:**
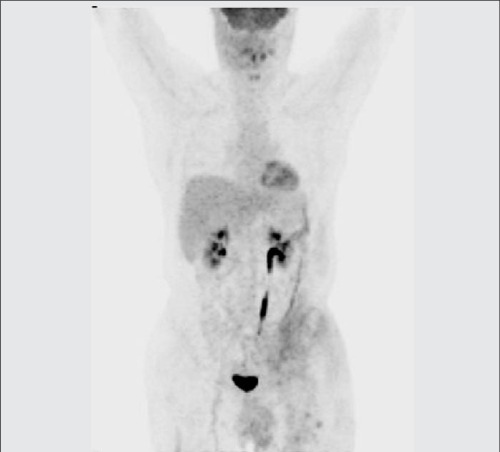
MIP whole body image showed non-homogeneous FDGuptake (SUV_max_:3.0) on the left lower side of anterior abdominalwall which was considered as secondary to the treatment
